# The underestimated danger of E-cigarettes - also in the absence of nicotine

**DOI:** 10.1186/s12931-018-0870-4

**Published:** 2018-08-29

**Authors:** Martina Korfei

**Affiliations:** 1 Department of Internal Medicine II, Klinikstrasse 36, 35392 Giessen, Germany; 2 Biomedical Research Center Seltersberg (BFS), Justus-Liebig-University Giessen, Schubertstrasse 81, 35392 Giessen, Germany; 3 Universities of Giessen and Marburg Lung Center (UGMLC), Member of the German Center for Lung Research (DZL), 35392 Giessen, Germany

Electronic cigarettes (E-cigarettes, ECs) are electronic devices that heat a liquid – usually comprising propylene glycol and glycerol, with or without nicotine and flavors, stored in disposable or refillable cartridges or a reservoir – into an aerosol (vapor) for inhalation [1]. Since ECs appeared on the market in 2006 they have become increasingly popular, especially among young people [1, 2]. E-cigarettes are marketed as a safer and “healthier” alternative to traditional cigarettes, and it is suggested in the mass media that ECs help smokers to stop smoking long-term, or to help smokers unable to stop smoking entirely to reduce their tobacco cigarette consumption [3]. However, the number of never-smoking youth who use ECs with or without nicotine is dramatically increasing. ECs are new sources of the highly addictive substance nicotine, which has a proven harmful effect on health [4, 5]. And, the advertising of nicotine-free ECs with liquids of fruit and sweet flavors is particularly likely to encourage young people to start using the E-cigarette. The Forum of International Respiratory Societies also revealed that E-cigarette (EC)-smoking is a significant public health problem because EC-use simulate smoking behaviour and can be done in public places, which together with its alarmingly growing popularity may increase the social approval for smoking and nicotine addiction [6].

In addition to the possible harmful effects of nicotine *per se*, the other main health concerns about EC-usage is the potential for toxic aldehyde emissions, such as formaldehyde, acetaldehyde, propanal and acrolein, which are known to be formed following heating of the EC-liquid main components propylene glycol and glycerol, as thermal decomposition products [7, 8]. Other contaminants inhaled by an EC-smoker during a “vaporization” session can be *o*-methyl-benzaldehyde, carcinogenic nitrosamines, terpenic compounds such as limonene (which are probably used by the manufacturers as flavoring agents) [9–11], as well as heavy metal and silicate particles (> 1 μm) including nanoparticles (< 100 nm) [12]. Interestingly, it has also been reported that the emission levels of aldehydes released by EC aerosols with and without flavorings, are generally very low [13, 14], and that high levels of formaldehyde emissions due to excessive degradation of propylene glycol can only be caused by unrealistic use conditions of ECs, such as overheating (remaining) faint levels of EC-liquids in the cartridges at a high voltage (5 V), which creates the unpleasant taste of “dry puffs” to EC-smokers and is thus normally avoided by users [13, 15, 16].

However, there is growing evidence from current studies suggesting that EC-smoking induces a signature of harm in the lung, which clearly challenges the concept that switching from traditional cigarettes to ECs is a healthier alternative. EC-smoke/EC-aerosol exposure has been demonstrated to induce oxidative stress, glutathione depletion and increased production of inflammatory cytokines in human airway epithelial cells in vitro and in lungs of mice in vivo [17]. Further, mice exposed to EC-smoke reveal impaired pulmonary anti-bacterial and anti-viral defenses in response to infection with *S. pneumoniae* and Influenza A virus, respectively [18]. These changes may play a role in the development of chronic airway diseases, such as chronic obstructive lung disease (COPD). In addition, DNA damage and impaired DNA repair mechanisms have recently been reported in human bronchial epithelial cells and in the mouse lung in response to EC-smoke exposure, suggesting enhanced susceptibility of the lung epithelium to oncogenic transformation and tumorigenesis [19]. In vivo evidence that EC-smoking/EC-aerosols can be harmful to the human lung stems from a recent study by Reidel et al. who compared 15 sputum samples from EC-users, 14 from current tobacco cigarette smokers and 15 from never-smokers by quantitative proteomics [20]. The underlying signature obtained from the sputum of EC-smokers is that of a unique innate immune response in the lung, involving increased neutrophilic activation and altered mucin secretion, as compared to never-smokers, and was in part overlapping with, but also distinct from that of healthy tobacco cigarette smokers to some extent. Additionally, signatures of increased reactive oxygen species (ROS) generation involving upregulation of aldehyde detoxification mechanisms were evident in both the EC-smokers and traditional cigarette smokers. However, the authors made it clear that most of the EC-smokers (12 of 15) were formerly tobacco cigarette smokers, thus questioning whether these results were solely related to EC-aerosols [20]. Therefore, studies designed to research EC-smokers without “former traditional cigarette-history” seem to be needed.

In their research article entitled “Altered lung biology of healthy never smokers following acute inhalation of E-cigarettes” in Volume 19 of the *Respiratory Research*-journal (Staudt et al.: *Respir Res* 2018, 19:78; [21]), Staudt and coworkers have researched the transcriptome in small airway epithelium (SAE) cells as well as in alveolar macrophages (AM) of healthy never-smoker indivduals with no history of exposure to any tobacco products or ECs (*n* = 10), versus SAE and AM obtained from the very same individuals after exposuring them to short-time EC-smoking in the absence (*n* = 3) or presence of nicotine (*n* = 7) in EC-aerosols. Short-time EC-smoking was defined by two exposures to 10 puffs with an interval of 30 min between both EC-usages. In their study, Staudt et al. could clearly demonstrate that short-time smoking of ECs is actually harmful to the lung, even in the absence of nicotine. They observed that the gene expression profiles were significantly altered in all conditions thereby indicating signatures of increased inflammation, impaired host-defense responses, p53-activation as well as pro-tumorigenic signaling relevant to lung cancer, as compared to the “never-smoking-state” before. In detail, the list of differentially regulated genes in supplemental Table IV of this paper [21] shows that SAE of individuals inhaling EC-smoke with nicotine indicated enhanced expression of the p53-activating tumor-suppressors *AJUBA* (Ajuba LIM protein) and *LATS2* (large tumor suppressor kinase-2) on the one side [22, 23], but simultaneously significant upregulation of well-known tumor and metastasis promoting factors, such as *SERPINB2* (=*PAI2*, plasminogen activator inhibitor-2) and *EDN1* (endothelin-1) on the other side [24, 25]. Notably, EC-smoking without nicotine (supplemental Table V in [21]) also resulted in significant activation of genes with a prominent role in promoting tumorigenesis, such as *BATF3* (basic leucine zipper transcription factor, ATF-like 3) [26], *S100P* (S100 calcium binding protein P) [27], *CEACAM5* (carcinoembryonic antigen-related cell adehesion molecule-5), and *FGFBP1* (fibroblast growth factor binding protein-1) [28]. FGFBP1 enhances FGF signaling including angiogenesis during cancer progression and is upregulated in various cancers [28], and *S100P* has been identified as target gene of the phosphoinositide 3-kinase/protein kinase B (PI3K/Akt) pathway in lung cancer cells, which is observed to be hyperactivated in most non-small cell lung cancers (NSCLCs) [27]. In line with this pro-tumorigenic signature, down-regulated genes included the circadian clock component *PER3* (period circadian clock 3), and loss of *PER3* has been suggested as a novel prognostic biomarker in patients with NSCLC [29]. Further, EC-smoking in the absence of nicotine led to upregulation of *LTB4R2* (leukotriene B4 receptor-2) and *ISG20* (interferon stimulated exonuclease gene-20 kDa) in SAE cells, which are indicative of increased inflammation and activated host innate immune signaling [30, 31]. Induction of expression and pro-inflammatory functions of LTB4R2 receptor have been demonstrated in bronchial epithelial cells in response to cigarette smoke extracts (CSE)-exposure in vitro, which promoted the adhesiveness of neutrophils to bronchial cells, a mechanism that has been suggested to contribute to airway neutrophilia and tissue damage in COPD in vivo [30]. Moreover, upregulation of the transcription factor *SPDEF* (SAM pointed domain-containing Ets transcription factor), a key positive regulator of the airway secretory mucin *MUC5AC* [32], suggests an elevated mucin concentration in SAE in response to EC-aerosol inhaling, and which indeed was observed in the study by Reidel B et al. in the sputum of EC-smokers [20]. It has been suggested that an elevated mucin concentration is an important hallmark of failed mucus transport in mucoobstructive disease and an important parameter in COPD pathogenesis [20, 33].

The AM transcriptome data of individuals inhaling EC-aerosols with nicotine (supplemental Table VI in [21]) indicated differentially regulated genes involved in host-pathogen interactions and immune-response, involving upregulated *ICAM4* (intercellular adhesion molecule 4) expression [34] and downregulation of *CCL28* chemokine, which has been reported to possess potent antimicrobial activity [35]. ICAM-4 has been reported to support host cell invasion by *M. tuberculosis* through direct binding of this pathogen [34], and its upregulation in response to nicotine-containing EC-smoke could indicate an increased susceptibility to bacterial invasion of macrophages in EC-users. Similar to the SAE transcriptome data, AM of EC-smokers inhaling nicotine-free aerosols (supplemental Table VII in [21]) also indicated a protumorigenic signature, as shown by increased expression of *PTGER3* [prostaglandin E_2_ (PGE_2_) receptor EP3 subtype] and *CCNB2* (cyclin B2) [36, 37]. Upregulated expression of *CCNB2* mRNA in tumor cells has been demonstrated to correlate with a poor prognosis in patients with NSCLC [37]. PGE_2_/EP3-receptor signaling has been reported to promote tumor growth in NSCLC through nuclear translocation of epidermal growth factor receptor (EGFR) and consequent up-regulation of cyclin D1 and c-Myc [36]. Increased PGE_2_/EP3-receptor signaling has also been observed to suppress lung innate immunity against *S. pneumoniae* [38], whereas *Ptger3* deletion improves pulmonary host defense and protects mice from death in severe *S. pneumoniae* infections or lipopolysaccharide (LPS) exposure [39]. Importantly, the signature of an increased susceptibility to respiratory bacterial infections was also supported by decreased expression of the immune response gene *ITGA6* (integrin alpha-6) [40] and the transcription factor *FOXM1* (Forkhead box protein M1), a critical mediator of lung development. It has been shown that Foxm1 regulates resolution of hyperoxic lung injury in neonatal mice, through inhibiting neutrophil-derived enzymes and enhancing monocytic responses that limit alveolar epithelial injury [41].

Taken together, these results by Staudt et al. unequivocally indicate that even short-time EC-smoking dysregulates biology of the human lung in vivo, independently of nicotine, and that inhaling of the non-nicotine derived chemicals present in EC-aerosols are actually harmful to SAE cells and AMs in EC-smokers, despite the limited and “correct” use of EC-devices (with avoiding “dry puffs”). As outlined, the observed gene expression changes in response to EC-aerosol exposure may have significant implications for lung tissue injury responses. Moreover, the transcriptome data are very well presented and will be a reference for other scientists looking at affected genes for studying the potential adverse effects of ECs. Though, it should be considered, that only one brand, namely “Blu EC” with and without nicotine, was analyzed in the study by Staudt and coworkers [21]. But the types or concentrations of chemicals a person is exposed to is varying by brand and type of device; and no-one can yet really estimate the harms of chemical reaction products formed in EC-aerosols from the plenty of merchandised EC-liquids with various flavoring compounds, which are thousands today and which differ by brand and manufacturer.

## Conclusion

In conclusion, the results of the study by Staudt et al. expand our insights about adverse effects of ECs, and at the very least, suggest that quoting ECs being considerably less harmful to health as compared to traditional cigarettes should be avoided until complete data on the safety and health impact of EC-smoking and various EC-aerosols are available. Due to lack of these informations, the Forum of International Respiratory Societies advances the view, that ECs should be restricted or banned, which is reasonable [6].

## Abbreviations

*AJUBA*: Ajuba LIM protein
AM: Alveolar macrophages
*BATF3*: Basic leucine zipper transcription factor, ATF-like 3
*CCL28*: C-C motif chemokine 28
*CCNB2*: Cyclin B2
*CEACAM5*: Carcinoembryonic antigen-related cell adhesion molecule-5
COPD: Chronic obstructive lung disease
CSE: Cigarette smoke extracts
EC(s): E-cigarette(s), electronic cigarette(s)
*EDN1*: Endothelin-1
EGFR: Epidermal growth factor receptor
*FGFBP1*: Fibroblast growth factor binding protein-1
*FOXM1*: Forkhead box protein M1
*ICAM4*: Intercellular adhesion molecule 4
*ISG20*: Interferon stimulated exonuclease gene-20 kDa
*ITGA6*: Integrin alpha-6
*LATS2*: Large tumor suppressor kinase-2
LPS: Lipopolysaccharide
*LTB4R2*: Leukotriene B4 receptor-2
*M. tuberculosis*: Mycobacterium tuberculosis
*MUC5AC*: Mucin-5 AC
NSCLC: Non-small cell lung cancers
*PER3*: Period circadian clock 3
PI3K/Akt: Phosphoinositide 3-kinase/protein kinase B
*PTGER3*: Prostaglandin E_2_ (PGE_2_) receptor EP3 subtype
ROS: Reactive oxygen species
*S. pneumoniae*: Streptococcus pneumoniae
*S100P*: S100 calcium binding protein P
SAE: Small airway epithelium
*SERPINB2* = *PAI2*: Plasminogen activator inhibitor-2
*SPDEF*: SAM pointed domain-containing Ets transcription factor
